# Icariin: a Potential Compound for the Recovery of Tibial Dyschondroplasia Affected Chicken Via Up-Regulating BMP-2 Expression

**DOI:** 10.1186/s12575-018-0080-y

**Published:** 2018-07-01

**Authors:** Mujahid Iqbal, Hui Zhang, Khalid Mehmood, Aoyun Li, Xiong Jiang, Yaping Wang, Jialu Zhang, Muhammad Kashif Iqbal, Mujeeb Ur Rehman, Wangyuan Yao, Shijin Yang, Jiakui Li

**Affiliations:** 1 College of Veterinary Medicine, Huazhong Agricultural University, Wuhan, 430070 People’s Republic of China; 2 University College of Veterinary and Animal Sciences, The Islamia University of Bahawalpur, Punjab, Pakistan; 3 College of Animal Husbandry and Veterinary Medicine, Tibet Agricultural and Animal Husbandry University, Linzhi, Tibet 860000 People’s Republic of China

**Keywords:** Chicken bone diseases, Tibial dyschondroplasia, Icariin, BMP-2, Gene expression

## Abstract

**Background:**

Tibial dyschondroplasia (TD) is a skeletal disease of fast growing chicken and other avian species. It is characterized by an avascular and non-mineralized growth plate, which leads to a deformed tibial bone and lameness. Unfortunately, this disease is not only responsible for causing huge economic losses but also raises animal welfare concerns. Icariin is a flavonoid, which is isolated from *Epimedium pubescens* herb, and it has been used to cure different diseases including bone fractures and osteoporosis.

**Results:**

We designed this experiment to use icariin for the treatment of TD affect chickens; for this purpose, a total of 180 chicks were equally divided into three groups: control, TD and icariin. All the three groups were offered ad libitum same normal standard diet with an addition of thiram (50 mg/kg) from 3rd day to 7th day in TD and icariin group in order to induce TD in chickens. After the induction of TD, the chickens in icariin groups were fed standard diet with an addition of icariin at the rate of 10 mg/kg in drinking water to check the therapeutic effect of this flavonoid on TD. Our results showed that the icariin helped in restoring the TD lesion into a normal structure with significantly (*P* < 0.05) up-regulating the bone morphogenetic protein-2 (BMP-2) expression in the tibial growth plates (GP).

**Conclusions:**

Icariin increased the vascular area in the growth plate and decreased the average TD score. In conclusion, this study shows that icariin is a potential compound for the recovery of TD affected chickens via up-regulating the BMP-2 expression without posing a threat of ingestion of toxic veterinary drug residues to human beings upon the consumption of treated chickens.

## Background

**Fig. 1:**
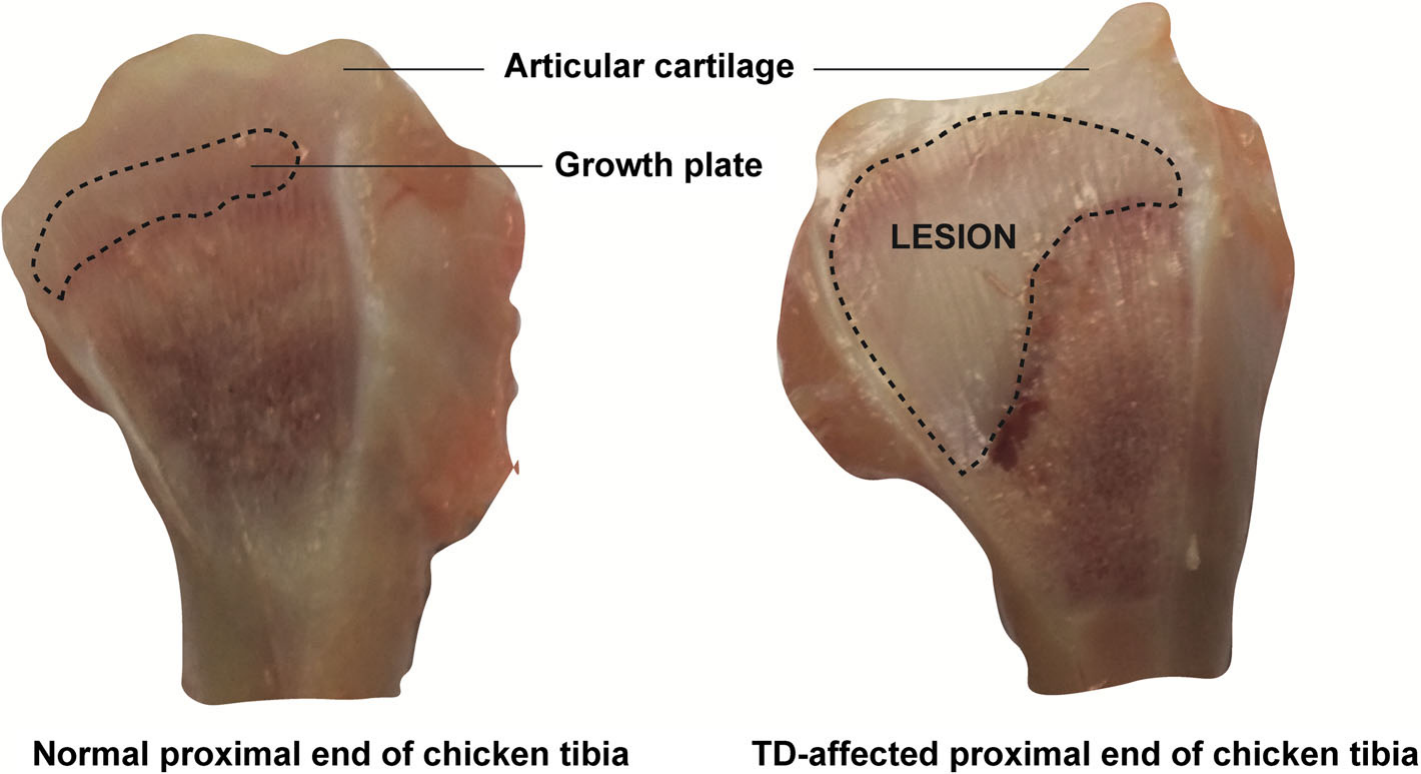
A visual difference between the normal and TD affected tibial bone of chicken

Tibial dyschondroplasia (TD) is the among the most common leg pathology of meat-type poultry. A typical lesion of TD is a mass of non-vascularized growth plate (GP) cartilage (Fig. 1) at the proximal tibiotarsus and tarsometatarsus. Generally, it is believed that TD is a result of maturing chondrocytes which fail to go thorough terminal differentiation [1–5]. In mammals, osteochondrosis is a disease which resembles TD [5].

Bone morphogenetic proteins (BMPs) belong to the transforming growth factor-β (TGF-β) superfamily and are responsible for the regulation of bone morphogenesis. These proteins have been associated to the differentiation and regeneration of bone [6, 7]. Recently, several studies have shown BMPs potentially regulate bone remodeling upon activating BMP signals in mice [8]. Moreover, BMP-2 is a known stimulator of bone-formation and osteoblastic differentiation [9]. Previously it has been reported, icariin up-regulate the BMP-2 expression in osteoblasts [10]. Hsieh et al. [9] found that, icariin contain the osteogenic properties via BMP-2/Smad pathway and nitric oxide (NO) synthesis. Nitric oxide regulates the Runx2 gene expression, which further contribute to the proliferation and differentiation of osteoblasts and BMP-2/Smad pathway suppresses caspase-3; thus, restrict the apoptosis of osteoblasts. Liang et al. [7] stated that icariin enhances the bone formation via BMP-2/Smad4 transduction pathway in human osteoblastic cell line.

Icariin (C_33_H_40_O_15_) is the main active compound of *Epimedium pubescens*, a Chinese herb recorded as “Yinyanghuo” in the Chinese pharmacopoeia. Chinese people have been using this medicine for decades to treat osteoporosis and bone fractures and to strengthen tendons [11, 12]. Previously, it has been reported that icariin can potentially repair bone and enhance bone formation. In addition to this, icariin has also been found to enhance bone regeneration [13, 14]. Several studies suggest the chondroprotective effects of icariin via promoting chondrogenesis and reducing the destruction of chondrocytes [15–17]. Therefore, in this study, we hypothesized that icariin is a potential compound to restore TD affected growth plate.

## Methods

### Experimental Design

A total of 180, one-day-old Arbor Acres chickens with an average weight of 47 ± 0.5 g were obtained from a local hatchery (Chia Tai Animal Husbandry Co. Ltd., Wuhan). The chickens were divided equally into 3 groups (60 chickens each groups). Later, the groups were named as control, TD, and icariin and were offered ad libitum diet following the National Research Council (NRC, 1994) guidelines. All the groups were fed the same standard Vitamin D rich diet to prevent rickets. From day 4 to day 7 tetramethylthiuram disulphide (thiram) at the rate of 50 mg/kg of feed was offered in TD and icariin group to induce tibial dyschondroplasia [18, 19]. After day 7, thiram was stopped in both groups and icariin was offered from day 8 to day 18 in the icariin group at the rate of 10 mg/kg/day to check the therapeutic effect this medicine on TD.

### Sample Collection and Handling

The chickens were raised for 18 days and during this period lameness was recorded on a daily basis. Randomly ten chickens from each group were slaughtered by cervical dislocation on day 7, 10, 14 and 18. The severity of TD lesion was determined according to Simsa et al. [20] and Pines et al. [21]. In short, normal growth plate was denoted as 0 score, recognizable plug of cartilage in the longitudinal section of the proximal end of tibia was denoted as 1 score, 20% area covered by the lesion was denoted as 2 score, 50% area covered by the lesion was denoted as 3 score and 80% area covered by the lesion was indicated as 4 score. An average score for each group was obtained by dividing the total score of all tibial bones in that group with total number of tibial bones. Immediately after TD scoring, tibial bones were stored in 4% paraformaldehyde for hematoxylin and eosin (H&E) staining and few of them were stored in liquid nitrogen at − 70 °C for Western blotting and reverse transcription quantitative real-time polymerase chain reaction (RT-qPCR) analysis.

### Hematoxylin and Eosin (H&E) Staining and Immunohistochemistry (IHC)

H&E staining of the proximal end of tibia bone was performed in order to observe normal structures in control group, damaged structures in TD group and to observe the extent of recovery in icariin group. Vascular area in growth plate was measured using Image-Pro Plus software by assigning different color codes to blood vessels and the rest of GP. The tibiotarsal bone samples stored in paraformaldehyde were exposed to 10% ethylenediamine tetra acetic acid for decalcification. After that, cleaning of bone samples was done by xylene and were embedded in paraffin wax. Subsequently, a 4–5 μm thickness sections were cut to prepare histological slides. H&E staining was performed according to a previously described method with slight modifications [22] and IHC was done according to an earlier reported method [23]. Several previous studies has demonstrated that BMP-2 are expressed in chondrocytes of GP, and may play an important role in their differentiation [24]. Therefore, we evaluated the BMP-2 antibody expression in GP via immunohistochemistry. The slides were washed in phosphate-buffered saline (PBS) and peroxidase-blocking solution (Boster, Wuhan). After that, the slides were incubated with 1:2000 dilutions of primary antibody against BMP-2 (ABclonal technology, Wuhan) at 4 °C overnight. Later the slides were washed using PBS and were incubated at 37 °C for 1.5 h with horseradish peroxidase-conjugated anti-rabbit secondary antibodies (Tuojie biological technology Co. Ltd., Wuhan). These immunolabeled slides were then examined under the microscope (Olympus CX31, Japan) and at last, primary antibodies were removed from negative control.

### RNA Extraction and RT-qPCR

GP tissue from each group was separated and homogenized using TRIzol reagent (Invitrogen, Carlsbad, CA, USA) for the extraction of total RNA, which was transcribed into cDNA using a First-Strand cDNA synthesis kit (Tian Gen, China). Primers for BMP-2 were designed by using Primer Premier Software and were synthesized by Wuhan Qingke biotech. Co. Ltd. (Wuhan) based on the sequences published in GenBank database. The reaction mixture was normalized against the reference gene GAPDH. All the RT-qPCR reactions were performed in quadruplex using Step One-Plus™ RT-qPCR system (Applied Biosystems). RT-qPCR reaction system was composed of 10 μL SYBR reaction mix (Transgen Biotech) forward and reverse primers of a working concentration of 10 μL mol/L, 2 μL of cDNA and nuclease free water for a total volume of 20 μL. All the reactions were performed with the following thermal cycling parameters: 95 °C for 30 s (sec), 40 amplification cycles at 95 °C for 8 s, 59 °C for a time of 30 s and 70 °C for 30 s. Relative quantification was performed using delta (ΔCt) method [25].

### Western Blot Analysis

GP was homogenized in ice-cold PBS, stored at 4 °C for 2 h. Later, the supernatant was collected after the centrifugation at 14,000 x g for 10 min. Total protein concentration was determined by BCA protein detection kit (Service Biotechnology, Wuhan, China) and the samples were stored at − 70 °C. Protein was separated by sodium dodecyl sulfate polyacrylamide gel electrophoresis (SDS-PAGE) until the dye band reached the end of gel (10% polyacrylamide gel) and was transferred on polyvinylidene difluoride (PVDF) membrane. These membranes were incubated in 5% skimmed milk for 1.5 h at room temperature. Subsequently, the membranes were incubated at 4 °C for overnight with rabbit monoclonal anti-BMP-2 primary antibodies (1:1000 dilution). Membranes were washed with tris-buffred saline tween (TBST) with a time span of 5 min for each time. Membranes were incubated at room temperature for 30 min with secondary antibody (1:3000 dilution) and were washed again with TBST 4 times. Finally, after washing the images were taken using an imaging system (UVP, Upland, CA, USA).

### Statistics

Data was examined by a one-way analysis of variance (ANOVA) and student t-test using SPSS software (version 19.0) and presented as means±standard error of means (S.E.M). The difference between control and treatment groups was considered significant if *P* < 0.05.

## Results

### Morphological Observations

**Fig. 2:**
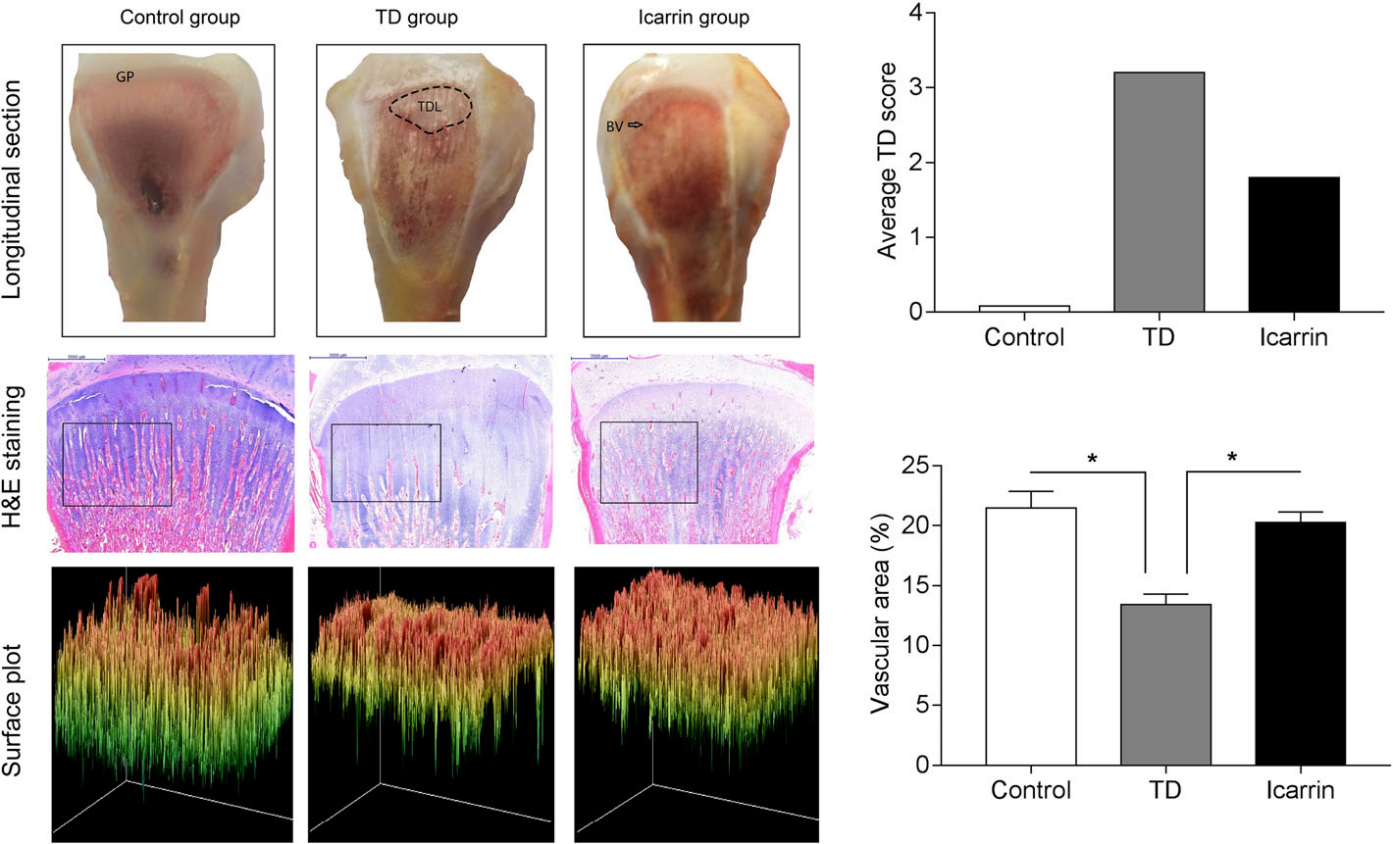
The morphological changes of growth plates in the proximal end of tibia (Longitudinal section and H & E staining) on day 18. Average TD score graph on day 18 (Upper graph). Surface plot of trabecular bone was generated and analyzed by Image-Pro Plus. Vascular area on day 18 (Lower graph) showing the percentage of blood vessel in different groups. *P < 0.05

As the thiram was started on the day 4 to induce TD, the chickens started showing typical signs of TD and associated lameness. The possibility of rickets was ruled out by feeding vitamin D rich diet. It was observed that lameness because of TD increased from day 4 to day 10 with a notable decline afterwards in TD group as the thiram was removed. Lameness in icariin group (treatment group) was less as compared to the TD group and based on morphological and histological observation it was observed that TD was restored largely (Fig. 2).

An average TD score on day 18 has been presented in Fig. 2. Average TD score in icariin group was non-significantly different on day 10 and 14, however, a gradual decrease in TD score was observed on day 10 and 14. Therefore, chickens started regaining the ability to stand and walk properly after the icariin administration. The physical appearance and the walking ability of chickens were almost similar as compared to the control group on day 18 after the continuous administration of icariin. There were no signs of lameness in control group throughout the experiment.

### Effect of Icariin on the GP of Thiram Induced TD

Histopathological observations showed, a mass of un-vascularized nature was present at the proximal end of tibia with a prominent cell death or apoptosis, irregular chondrocytes and degradation in thiram fed chickens (TD group) on day 7, 10, 14 and 18. While the control group showed a normal GP with regular chondrocytes and columns. Large number of blood vessel were also observed in proliferation and hypertrophic zone in control group. However, icariin group showed a gradual improvement after the administration of icariin on day 10, 14 and 18. Icariin treatment restored the blood vessel formation and chondrocytes differentiation in GP (Fig. 2).

### Immunohistochemical Localization of BMP-2 and Protein Expression in GP

**Fig. 3:**
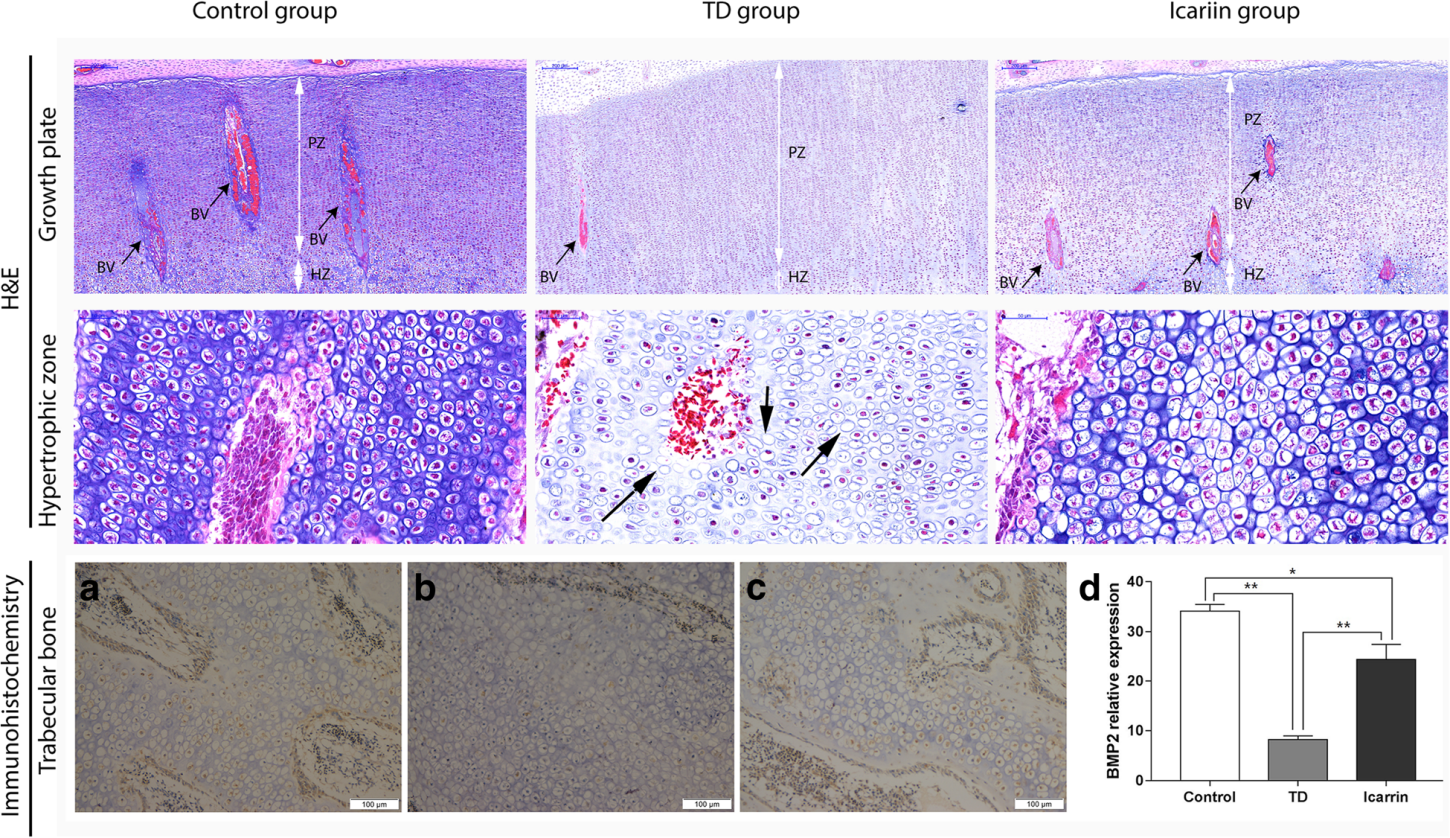
The hematoxylin and eosin (H&E) staining of growth plate (PZ = Prehypertrophic zone, HZ = Hypertrophic zone, BV = Blood vessels) and hypertrophic zone (Large arrows = cell death) of tibia and Immunohistochemical localization of BMP-2 in control (**a**) TD (**b**) and icariin (**c**) group. Graph (**d**) shows the BMP-2 relative expression in all the three groups on day 18. *P < 0.05

**Fig. 4:**
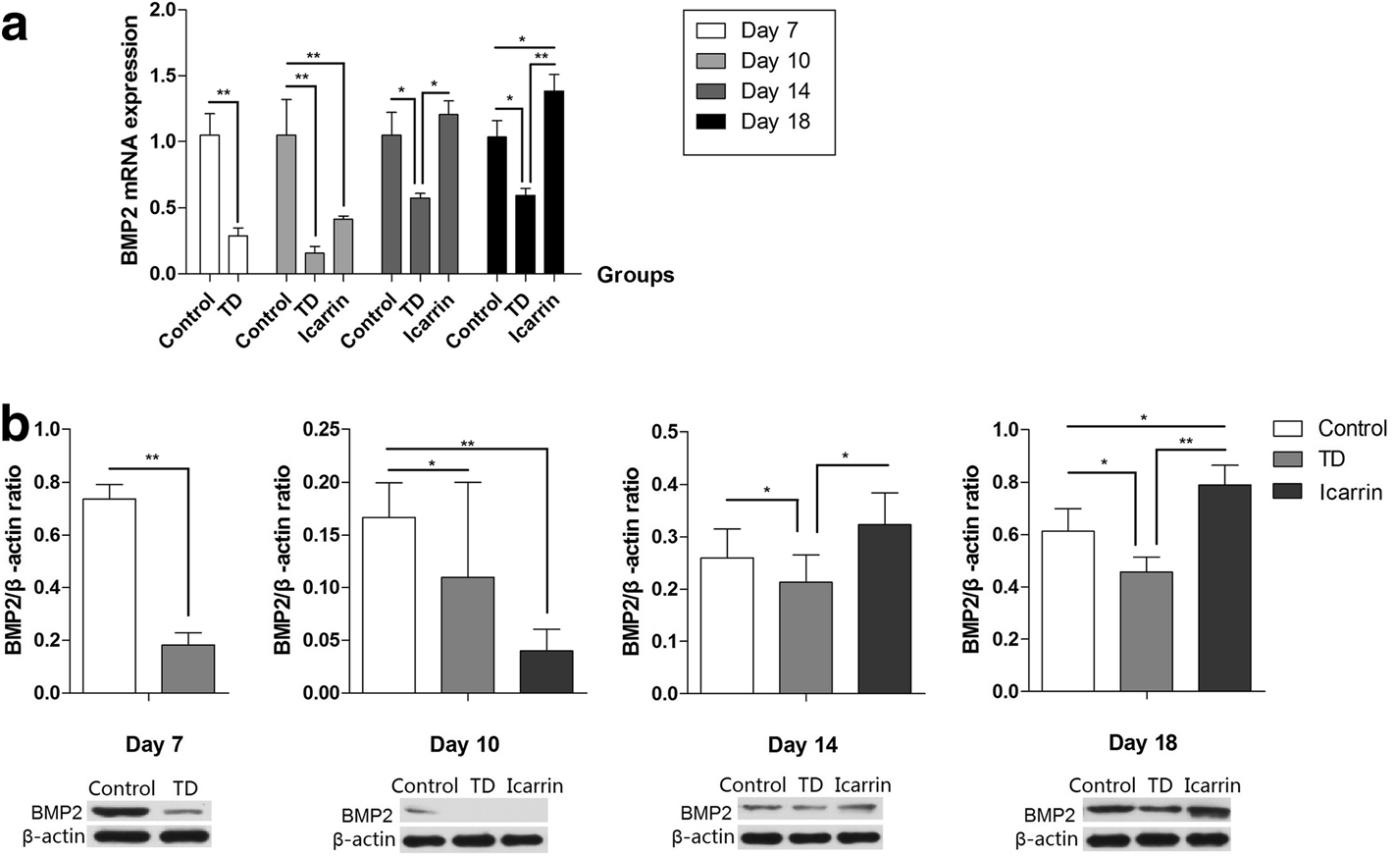
Real-time quantitative PCR analysis and protein levels of BMP-2 were analyzed in growth plate on day 7, 10, 14 and 18. **a** The mRNA levels of BMP-2 was detected by RT-qPCR; **b** The protein levels of BMP-2 was detected by Western blotting. Western blot bands were quantified by ImageJ®. *P < 0.05, **P < 0.01

Immunohistochemical results showed that a less number of cells localized BMP-2 in TD suffering chickens as compared to the control and icariin treated group (Fig. 3). On day 18, BMP-2 expression was significantly decreased (*P* < 0.01) in TD group as compared to the control group. While, icariin administration significantly (*P* < 0.01) up regulated the expression of BMP-2 in the GP of treated chickens (Fig. 4a) as compared to the TD group. Protein level was determined by Western blot analysis on the day 7, 10, 14 and 18 in tibia bone of each group. The level of BMP-2 protein was significantly (*P* < 0.01) down regulated on day 7 and onward in TD group while it was significantly (*P* < 0.01) increased after the treatment with Icariin on day 18 (Fig. 4b).

## Discussion

Poultry is major source of protein and the demand of poultry meat and other by-products are increasing day-by-day throughout the world [26]. Among other poultry diseases, skeletal problems are a major cause of economic losses [27]. Tibial dyschondroplasia is a metabolic disease of cartilage in the growth plate of chicken and other poultry species. TD is causing economic losses to the poultry industry by low yield and poor quality of meat [3]. It is believed that TD is caused by several reasons in fast growing chickens. However, recent research has found that bone reformation and remodeling play a key role in TD pathogenesis. TD lesion is characterized by an uneven white, non-mineralized and avascular mass of cartilage in the GP. While a normal avian GP shows regularly arranged long chondrocytes with plenty of blood vessels [1–3, 22, 28]. Chickens suffering from TD usually show difficulty in standing and walking, which ultimately leads to less feed intake and sometimes even death [22, 28, 29]. Our results in this study were also in accordance to the previously observed clinical symptoms. However, the icariin administration to the thiram-induced TD chickens significantly reduced the lameness by reducing the TD lesion and by improving the vascularization of chicken growth plate.

Chinese herbs are considered economical and safe because of their less toxicity [30]. Icariin is a flavonoid, which is isolated from *Epimedium pubescens* herb, and it has been used to cure different diseases including bone fractures and osteoporosis [31]. It promotes the bone formation and significantly increases the mineral contents and bone mineral density [7, 10]. The reason behind the wide use of icariin as a potential alternative therapy for bone diseases is the capability of icariin to promote osteoblast proliferation and osteogenic differentiation [12, 32]. However, to the best of our knowledge no previous studies used this medicine to treat TD in chickens in relation to BMP-2.

In this experiment, thiram (fungicide) was used for the induction of TD. Thiram has been known for causing TD in chicken and other poultry species [1, 2] Excess growth was present in tightly arranged immature cartilage cells in TD group on day 7, 10, 14 and 18; there were scarce blood vessels in proliferation zone. Apoptosis and cell death was observed in the histopahological slides of severe TD lesions (Fig. 3). However, after treating the TD affected group with icariin the chickens regained the normal tibial structure and physically showed an ability to stand and walk properly.

BMPs play an essential role in the maintenance and repair of bone [33]. Recent studies show that icariin has been used to treat and understand the recovery mechanisms in bone related issues via regulating BMP-2. Icariin promote the bone formation via BMP-2/Smad pathway and nitric oxide synthesis [9]. As, BMP-2, along with other BMPs belong to TGF-β superfamily that stimulate the bone and cartilage formation. In this regard, Yin et al. [10] reported that icariin can significantly increase the proliferation and differentiation of osteoblasts in human via up-regulating the BMP-2 expression. Similar findings were published in another recent study, Zhang et al. [34] found that flavonoids of *Herba Epimedii* (icariin) can promote the osteogenic differentiation through the increased mRNA expression of BMP-2 in human bone marrow-derived mesenchymal stem cells. In another in vitro study, icariin was also shown to stimulate the MC3T3-E1 differentiation by inducing BMP-2 expression [8]. In this experiment, icariin significantly up-regulated the BMP-2 expression on day 14 and 18 in diseased chicken. Our results regarding the effect of icariin on BMP-2 expression were similar to above the mentioned studies and various other studies [7, 35].

## Conclusions

We conclude that icariin is a potential compound in promoting the angiogenesis in TD affected growth plates and it can significantly increase the BMP-2 expression. Considering the economic losses caused by TD and the cost of Icariin. We believe, icariin is a valuable alternative therapeutic agent for TD and deserves further research attention.

## Abbreviations

BMP-2: Bone morphogenetic protein-2
GP: Growth plates
H&E: Hematoxylin and eosin
IHC: Immunohistochemistry
NO: Nitric oxide
PBS: Phosphate-buffered saline
PVDF: Polyvinylidene difluoride
RT-qPCR: Reverse transcription quantitative real-time polymerase chain reaction
SDS-PAGE: Sodium dodecyl sulfate polyacrylamide gel electrophoresis
TBST: Tris-buffred saline tween
TCMs: Traditional Chinese medicines
TD: Tibial dyschondroplasia
TGF-β: Transforming growth factor-β
